# Genetic Deletion of Menin in Mouse Mesenchymal Stem Cells: An Experimental and Computational Analysis

**DOI:** 10.1002/jbm4.10622

**Published:** 2022-04-07

**Authors:** Jad Abi‐Rafeh, Meisam Asgari, Ildi Troka, Lucie Canaff, Ahmed Moussa, Damiano Pasini, David Goltzman

**Affiliations:** ^1^ Department of Medicine McGill University and McGill University Health Centre Montreal Canada; ^2^ Department of Mechanical Engineering McGill University Montreal Canada; ^3^ Theoretical and Applied Mechanics Program, School of Engineering and Applied Science Northwestern University Evanston IL USA

**Keywords:** BIOMECHANICS, BONE REMODELING, GENETIC MOUSE MODEL, MENIN AND BONE, MESENCHYMAL STROMAL/STEM CELLS

## Abstract

Loss‐of‐function mutations in the *MEN1* tumor‐suppressor gene cause the multiple endocrine neoplasia type 1 syndrome. Menin, the *MEN1* gene product, is expressed in many tissues, including bone, where its function remains elusive. We conditionally inactivated menin in mesenchymal stem cells (MSCs) using paired‐related homeobox 1 (Prx1)‐Cre and compared resultant skeletal phenotypes of *Prx1‐Cre*;*Men1* ^*f/f*^ menin‐knockout mice (KO) and wild‐type controls using in vivo and in vitro experimental approaches and mechanics simulation. Dual‐energy X‐ray absorptiometry demonstrated significantly reduced bone mineral density, and 3‐dimensional micro‐CT imaging revealed a decrease in trabecular bone volume, altered trabecular structure, and an increase in trabecular separation in KO mice at 6 and 9 months of age. Numbers of osteoblasts were unaltered, and dynamic histomorphometry demonstrated unaltered bone formation; however, osteoclast number and activity and receptor activator of NF‐κB ligand/osteoprotegerin (RANKL/OPG) mRNA profiles were increased, supporting increased osteoclastogenesis and bone resorption. In vitro, proliferative capabilities of bone marrow stem cells and differentiation of osteoblasts and mineralization were unaltered; however, osteoclast generation was increased. Gross femur geometrical alterations observed included significant reductions in length and in mid‐metaphyseal cross‐sectional area. Atomic force microscopy demonstrated significant decreases in elasticity of both cortical and trabecular bone at the nanoscale, whereas three‐point bending tests demonstrated a 30% reduction in bone stiffness; finite element analysis showed morphological changes of the femur microgeometry and a significantly diminished femur flexural rigidity. The biomechanical results demonstrated the detrimental outcome of the accelerated osteoclastic bone resorption. Our studies have a twofold implication; first, *MEN1* deletion from MSCs can negatively regulate bone mass and bone biomechanics, and second, the experimental and computational biomechanical analyses employed in the present study should be applicable for improved phenotypic characterization of murine bone. Furthermore, our findings of critical menin function in bone may underpin the more severe skeletal phenotype found in hyperparathyroidism associated with loss‐of‐function of the *MEN1* gene. © 2022 The Authors. *JBMR Plus* published by Wiley Periodicals LLC on behalf of American Society for Bone and Mineral Research.

## Graphical abstract

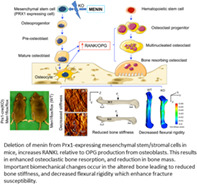

## Introduction

The autosomal dominant multiple endocrine neoplasia type 1 (MEN1) syndrome in which tumors arise in select endocrine tissues, including the parathyroid glands, and in non‐endocrine tissues is caused by loss‐of‐function mutations in the tumor suppressor gene, *MEN1*.^(^ ^1^, ^2^ ^)^ The *MEN1* gene product, menin, is widely expressed from early in fetal development and onward.^(^ ^3^, ^4^, ^5^, ^6^ ^)^ In various endocrine cell lines, menin has been shown to directly bind to Smad3 and mediate the cell proliferation and differentiation actions of transforming growth factor‐β (TGF‐β).^(^ ^7^, ^8^, ^9^ ^)^ In bone cell lines, menin interacts physically and functionally with bone morphogenic protein‐2 (BMP‐2), Smads1/5, and Runx2 to promote mesenchymal stem cell commitment into the osteoblast lineage, and with TGF‐β/Smad3 to maintain osteoblast function and differentiation.^(^ ^10^, ^11^, ^12^ ^)^

Heterozygous (*Men1* ^+/−^) global knockout mice develop tumors in the pituitary, pancreas, and parathyroid glands at 9 months of age, closely mimicking the human MEN1 disorder.^(^ ^13^, ^14^, ^15^ ^)^ Homozygous null (*Men1* ^−/−^) mice die in utero at mid‐gestation from development failure of multiple organs. The fetuses exhibit craniofacial defects, suggesting that menin may be critical for bone development in vivo.^(^ ^13^, ^14^ ^)^ Conditional knockout in mice of the *MEN1* gene in Pax3‐ or Wnt1‐expressing cells of the neural crest (which are precursors of mesenchymal stem cells) leads to perinatal death with cleft palates and other craniofacial defects.^(^ ^16^ ^)^ We have previously shown, using an *Osteocalcin* (*OC)‐Cre*‐mediated recombination strategy to conditionally disrupt the *MEN1* gene in mature osteoblasts, that menin was required for optimal bone formation and maintenance of bone mass.^(^ ^17^ ^)^ Deletion of menin in early osteoblasts and osteocytes using the osteoblast transcription factor Runx2 and the DMP1 (dentin matrix acidic phosphoprotein 1) promoters revealed a role for menin in controlling osteoclastogenesis via the osteoblast lineage.^(^ ^18^ ^)^

In recent years, there has been growing interest in molecular mediators of bone pathways to identify novel therapeutic targets for low bone mass disorders such as osteoporosis. Menin has emerged as one such potential therapeutic target.^(^ ^17^ ^)^

In mesemchymal stem cells, represented by the bone marrow cell line ST2, in vitro knockdown of menin expression achieved by transfection of menin antisense cDNA resulted in decreased bone morphogenetic protein (BMP)‐2‐induced alkaline phosphatase activity and osteocalcin and Runx2 mRNA expression. Menin was co‐immunoprecipitated with Smad1/5 and inactivation of menin antagonized BMP‐2‐induced transcriptional activity of Smad1/5 in ST2 cells but not in more mature MC3T3‐E1 osteoblastic cells. Menin also co‐immunoprecipitated with the key osteoblast regulator, Runx2, and anti‐sense menin diminished Runx2 transcriptional activity and the ability of Runx2 to stimulate alkaline phosphatase activity only in ST2 cells.^(^ ^11^ ^)^ These studies suggested that deletion of menin in mesenchymal cell precursors may produce distinctive actions from deletion in more mature cells of the osteoblast lineage.

In the present study, we therefore conditionally inactivated the *MEN1* gene in mouse MSCs in vivo using the Cre‐LoxP recombination system, and used high‐resolution imaging, and histological and biomechanical techniques to investigate the role of menin in the regulation of bone development in vivo. We demonstrate herein that menin functions critically in mesenchymal stem cells in vivo to regulate bone mass, bone structure, and bone mechanics, including microstructural and macroscopic geometric features as well as overall and local mechanical properties.

## Materials and Methods

### Generation of conditional knock‐out mice

A conditional knockout model in which the *Men1* gene is deleted in mesenchymal stem cells (MSCs) was generated. Mice were obtained from the Jackson Laboratory (Bar Harbor, ME, USA) and housed in a Canadian Council on Animal CARE (CCAC)‐accredited pathogen‐free standard animal facility at the Research Institute of McGill University. Mice were fed normal 18% protein chow diet (Envigo [TEKLAD global], Madison, WI, USA; product #T.2918.15) containing 1.0% calcium, 0.7% phosphorus, and 1.5 IU vitamin D_3_/g. All experimental procedures were performed following an animal use protocol approved by the Facility Animal Care Committee (FACC) of McGill University and met the ARRIVE guidelines. All personnel working with the mice were qualified and met all training requirements set by the FACC.

*Men1* ^*flox/flox*^ (129S(FVB)‐*Men1* ^*tm1.2Ctre*^/*J*) mice that possess loxP sites flanking exons 3–8 of the *Men1* gene were crossed with *Prx1‐Cre* ^*TG/+*^ transgenic mice (B6.Cg‐Tg(*Prrx1‐cre*)1Cjt/J), which express Cre recombinase in early limb bud and a subset of craniofacial mesenchymes.^(^ ^13^, ^19^ ^)^ The resulting *Prx1‐Cre* ^*TG/+*^; *Men1* ^*+/flox*^ mice were crossed with Men1^flox/flox^ mice to generate litters containing ~25% *Prx1‐Cre* ^*TG/+*^; *Men1* ^flox/flox^ mice, which were used for subsequent crosses with *Men1* ^*flox/flox*^ mice. The *Men1* ^*flox/flox*^ control mice are designated as wild type (WT), and *Prx1‐Cre*; *Men*1 ^*flox/flox*^ mice as knockout (KO). These mice are on a mixed FVB/C57BL/6J background. To examine parameters in heterozygotes (Het), *Prx1‐Cre*; *Men*1^*+/flox*^ male mice and female *Men1* ^*+/flox*^ mice were crossed, and resulting WT and heterozygote littermates were compared.

### Mouse genotyping and testing deletion of *Men1* exons 3–8 (Δ3–8)

**Fig. 1:**
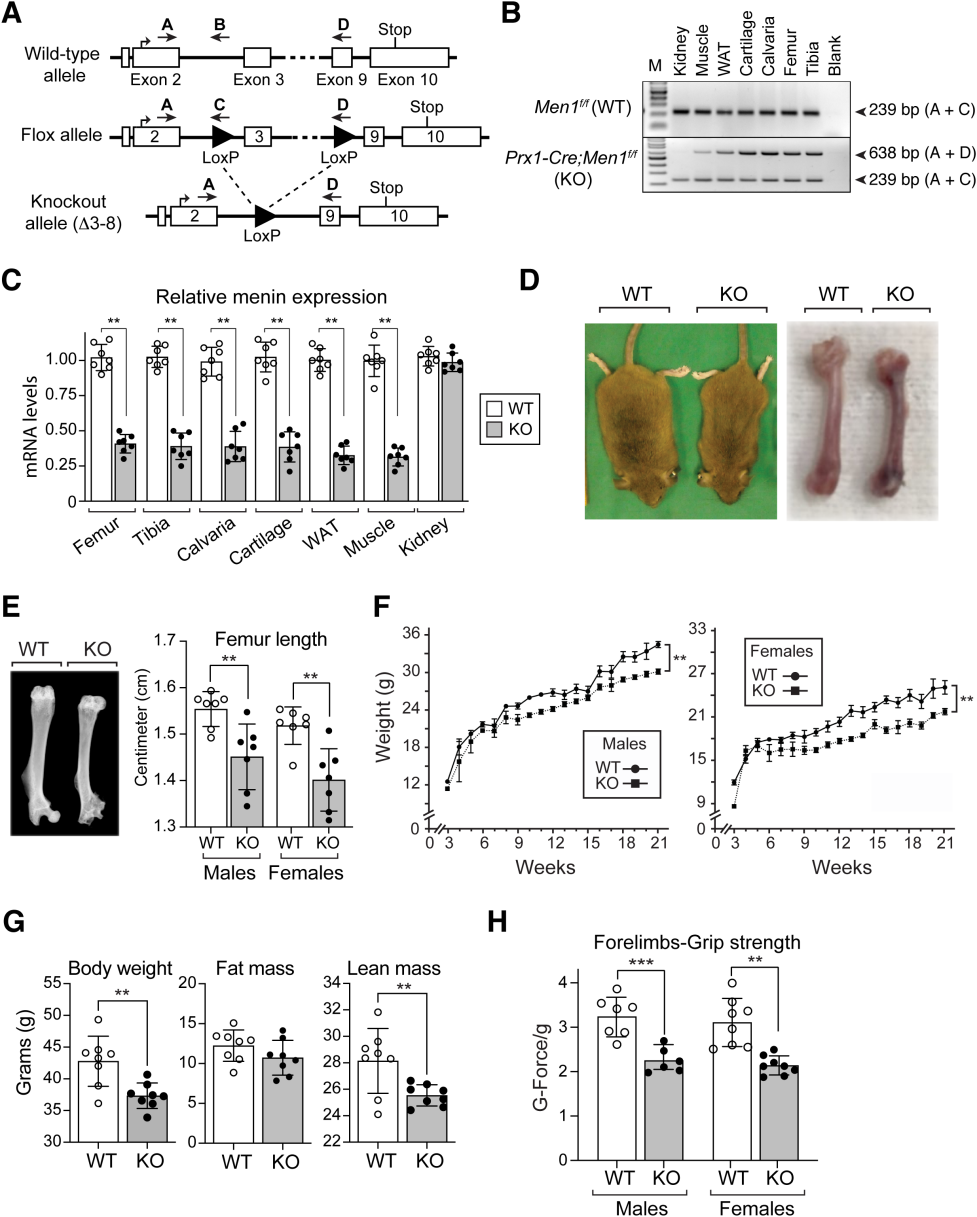
Global characterization of *Men1* ^f/f^ (WT) and Prx1‐Cre;*Men1* ^f/f^ (KO) mice. (*A*) In vivo manipulation of the *Men1* gene. The genomic structure of the wild‐type (WT) allele of the *Men1* gene (top) was altered (middle, Flox allele) by the insertion of LoxP sites (filled triangles) to generate the recombinant Δ3–8 *Men1* allele (bottom, knockout allele). Locations of the genotyping primers A, B, C, and D are indicated (arrows). (*B*) The *Men1* gene deletion is present in tissues of mesenchymal origin. Genomic DNA from WT and KO mouse tissues was used as a template for multiplex PCR amplification with primers A, C, and D. Product A + D = 638 bp (recombinant Δ3–8 *Men1* allele); product A + C = 239 bp (target *Men1flox* allele). (*C*) Gene expression levels of menin in WT and KO tissues determined by qPCR and normalized to GAPDH expression levels (*n* = 7). (*D*) Representative photograph of WT and KO male mice and dissected femurs at 9 months of age. (*E*) Representative femur X‐ray radiographs, and femur lengths of 6‐month‐old male and female WT and KO mice (*n* = 7). (*F*) Body weights of male (*n* = 8) and female (*n* = 7) WT and KO mice from 3 weeks to 21 weeks of age. (*G*) Echo‐MRI analysis of 12‐month‐old WT and KO male mice showing total body weight, fat mass, and lean mass (*n* = 8). (*H*) Grip strength of mouse forelimbs was measured, using a grip strength meter, in 6‐ to 7‐month‐old male (*n* = 6) and female (*n* = 7) mice, by allowing individual mice to grip a metal grid with only their forelimbs. Grip strength values (G‐Force) were normalized to body weight (g). Data are presented as mean ± SD; statistical significance indicated on plots: **p < 0.01, ***p <0.001.

Genomic DNA was extracted using the Quanta Bioscience (Beverly, MA, USA) DNA extraction kit (tail snips) or DNeasy Blood & Tissue Kit (Qiagen, Toronto, Canada) (other tissues). The 2× green PCR Master‐mix (ZmTech Scientifique, Montreal, Canada) was used for polymerase chain reactions (PCR). Mice were genotyped by PCR for the *Prx1*‐*cre* transgene with forward 5′‐CTAGGCCACAGAATTGAAAGATCT‐3′ and reverse 5′‐ GTAGGTGGAAATTCTAGCATCATCC‐3′ primers. Four primers were used to detect the presence of either wild‐type, floxed, or Δ3–8 *Men1* alleles (Fig. 1*A*): primer A (5′‐CCCACATCCAGTCCCTCTTCAGCT‐3′), specific to exon 2 of *Men1*; primer B (5′‐CCCTCTGGCTATTCAATGGCAGGG‐3′), specific to the wild‐type *Men1* sequence in intron 2, which is deleted by the cloning of the loxP sequence; primer C (5′‐CGGAGAAAGAGGTAATGAAATGGC‐3′), specific for the inserted loxP sequence, and primer D (5′‐CATAAAATCGCAGCAGGTGGGCAA‐3′), specific for *Men1* exon 9. PCR of genomic DNA generates amplicons of 300‐bp (wild‐type, primers A + B), 239‐bp (*Men1* ^*flox*^ allele, primers A + C), and 638‐bp (recombinant Δ3–8 *Men1* allele, primers A + D).

### Gene expression analysis

For gene analysis, quantitative reverse transcrition PCR (qRT‐PCR) was performed on RNA from long bones, calvaria, cartilage, white adipose tissue (WAT), muscle, and kidneys. Total RNA was extracted by the standard TRIzol method (Thermo Fisher Scientific Life Sciences, Waltham, MA, USA).

Briefly, tissues were collected from 6‐ to 7‐month‐old male and female mice and placed in RNAlater solution (Thermo Fisher‐Ambion, Carlsbad, CA, USA) for subsequent RNA isolation. Epiphyses and soft tissues were removed from bones and the marrow was flushed. Bones were crushed in liquid nitrogen and reconstituted inTRIzol reagent. Other tissues were homogenized in TRIzol reagent using the Brinkmann POLYTRON PT 300 Blade‐Type Homogenizer (Kinematica, Luzern, Switzerland), and subsequent steps were performed as per the manufacturer's instructions to isolate total RNA. Two micrograms of RNA was used for cDNA synthesis using the High‐Capacity cDNA Reverse Transcription Kit (Applied Biosystems, Foster City, CA, USA). Complementary DNA (cDNA) was diluted by a 1:1 ratio, and quantitative real‐time PCR (qPCR) performed thereafter on 3 μL of cDNA for each 20 μL reaction. Power SYBR Green PCR Master Mix and the ViiA 7 thermal cycler system machine were used for this step (Applied Biosystems); each sample was run in triplicate. Conditions for the reactions were as follows: 40 cycles: 95°C for 15 seconds, 56 to 58°C for 45 seconds, and 72°C for 60 seconds. GAPDH was used as the housekeeping gene reference, and expression levels were calculated using the ∆∆Ct method. To avoid amplification of genomic DNA, the primers chosen to amplify menin RNA crossed 2 introns: exon 2 forward primer 5′‐TTCCTGGCTGTCAACCGTG‐3′ and exon 4 reverse primer 5′‐TTCCTGGCTGTCAACCGTG‐3′. The housekeeping GAPDH primers used for normalization were: forward 5′‐CACCATCTTCCAGGAGCGAG‐3′ *GAPDH* reverse 5′‐CCTTCTCCATGGTGGTGAAG‐3′.

### Forelimb grip strength test

A forelimb grip strength test was used to assess changes in skeletal muscle strength in 6‐ to 7‐month‐old male and female wild‐type and knockout mice as previously described.^(^ ^20^ ^)^ Each mouse was lifted by its tail over the top of a grid so that it could grasp the grid platform with its front paws only. With its torso oriented in a horizontal position, the mouse was then gently pulled backward until it released the grid. The maximal force digitally applied by the mouse before releasing its grasp of the grid was displayed and recorded in grams on a grip strength meter (Bioseb, Chaville, France). The observer was blind to genotype and the mean of 10 consecutive tests per mouse was taken as an index of forelimb grip strength. The body weight of each individual mouse was determined at the end of the experiment to normalize the mean grip strength force to body weight.

### Echo‐MRI

Body composition of adult male mice was assessed at 12 months of age on live animals using the Echo‐MRI system (model ET‐040, version 11,06,22) at the McGill Mouse Metabolic Platform. Total body fat percentage was calculated as: [fat mass (g) / total body weight (g)] * 100% and total lean mass percentage as: [lean mass (g) / total bodyweight (g)] * 100%.

### Bone mineral density (BMD) analysis by dual‐energy X‐ray absorptiometry (DXA)

DXA analysis of 7‐ to 8‐month‐old male and female mouse femurs was performed at the Centre for Bone and Periodontal Research Core Facility at McGill University as previously described.^(^ ^17^ ^)^ Samples were placed prone on a specimen tray and the Kubtec Xpert 80 radiography system (KUB Technologies Inc., Milford, CT, USA) was used. Energy used was 21 kV/500 μA. Width/center used was 23862/4903 and magnification was 3×. BMD was calculated with the manufacturer's software.

### Micro‐computed tomography (micro‐CT)

Femurs from 7‐ to 9‐month‐old male and female mice were dissected free of soft tissue and frozen in PBS until analysis. High‐resolution images of the distal femur were acquired with a desktop microtomographic imaging system (SkyScan 1272, Antwerp, Belgium) in accordance with the recommendations of the American Society for Bone and Mineral Research (ASBMR).^(^ ^21^ ^)^

Images were captured at 45 kV and 222A, every 0.9° through a 180° rotation, using a detection pixel size of 5.625 μm, or every 0.9° through a 360° rotation, using a detection pixel size of 11.25 μm. Reconstruction of raw images was performed using NRecon software (SkyScan), while trabecular volumes of interest (VOI) were defined, modeled, and analyzed using the CTAn software (SkyScan). Trabecular regions were taken to include all cancellous bone in the metaphysis, defined within a 2 mm region that starts 1 mm below the growth plate. Three‐dimensional renderings of the distal 3.5 mm of each femur were created using the CTVox software (SkyScan).

### Histomorphometry

Static and dynamic histomorphometry were performed on undecalcified and decalcified femurs from 6‐ to 7‐month‐old male and female mice as previously described.^(^ ^17^ ^)^ Calcein (Sigma‐Aldrich, St. Louis, MO, USA; C‐0875) was injected intraperitoneally at a dose of 25 μg/g body weight on days 10 and 3 before euthanization, defined as day 0, when mice reached exactly 3 months of age. Mineral apposition rate (MAR) was defined as the average distance between two calcein fronts on a double‐label surface, divided by the time span between the two calcein doses (7 days). Mineralizing surface/bone surface (MS/BS) was assessed using the formula (dL + ½(sL) / BS, where dL and sL are the total double‐ and single‐labeled bone surfaces, respectively, and BS is the total bone surface in the analyzed field. Bone formation rate/bone surface (BFR/BS) was calculated as the product of the MAR and MS/BS measurements.

### Femur histology

Femurs from 3‐ and 7‐month‐old male and female mice were isolated and fixed in 4% paraformaldehyde solution and decalcified using 10% EDTA for 2 weeks. Samples were then dehydrated using a graded ethanol series and were paraffin‐embedded. Coronal sections (5 μm) were obtained using the Leica RM 2255 automated microtome (Leica Biosystems, Wetzlar, Germany), and stained for alkaline phosphatase (ALP) or tartrate‐resistant acid phosphatase (TRAP), for osteoblast and osteoclast visualization, respectively. High‐resolution slide scans were acquired using the Aperio At Turbo Digital Pathology Scanner (Leica Biosystems). For each randomly numbered sample, 3 to 5 sections were analyzed in full for bone cell numbers, at 4× magnification, using the ImageJ software (NIH). Undecalcified bones were embedded in methyl methacrylate (MMA), and 1‐μm sections were cut on an ultramicrotome. These sections were stained with the Goldner trichrome method as previously described.^(^ ^22^ ^)^ All histological analyses were conducted as per the recommendations of the American Society for Bone and Mineral Research Histomorphometry Nomenclature Committee.^(^ ^23^ ^)^

### Serum markers of bone metabolism

Male and female mice (5 to 7 months old) were fasted for 12 hours and serum samples were obtained from blood collected by cardiac puncture immediately subsequent to euthanasia. Serum measurements of osteocalcin, procollagen 1 intact N‐terminal propeptide (P1NP), C‐telopeptide degradation products from type 1 collagen (CTx), and TRAP were performed using ELISA kits (Biomedical Technologies Inc., Stoughton, MA, USA, and Immunodiagnostic Systems (IDS), Bolton, UK, respectively), according to the manufacturer's intructions.

### Three‐point bending tests

Biomechanical testing was performed on femurs isolated from 6‐ and 9‐month‐old male mice using the Instron 5943 machine (Instron, High Wycombe, UK). A downward bending load was applied at a rate of 0.05 mm/s to the posterior midshaft with a supporting distance of 7 mm from each side. A load‐displacement curve was generated and used to measure biomechanical parameters such as stiffness (N/mm), ultimate force (N), ultimate displacement (mm), and work to ultimate fracture (N.mm). Young's modulus was defined as the mean value of the maximum slope of the elastic portion of the load‐displacement curve. The initial slope was disregarded because it is not representative of the femur response; rather it can be attributed to the testing apparatus and the unengaged specimen.

### Atomic force microscopy (AFM)

Femurs from 9‐month‐old male mice were dissected and embedded in methyl methacrylate. Longitudinal and transverse sections of 50 to 100 μm thickness were then cut using an ultramicrotome (Leica RM 2165) using a diamond blade, with minimum surface roughness of <5 nm for AFM imaging and indentation.^(^ ^24^, ^25^ ^)^

A JPK Atomic Force Microscope (NanoWizard3 BioScience, JPK, Berlin, Germany) was used for imaging and force spectroscopy. Indentation force measurements were made at representative points on the longitudinal and transverse sections. Super‐sharp standard force modulation mode reflex coating (FMR) cantilevers (Nanotools USA LLC, Henderson, NV, USA) with diamond‐like carbon nano‐tip (radius 2 to 3 nm, nominal resonance frequency 75 kHz, and length 225 μm) were used for contact mode imaging. For indentation measurements, non‐contact high resonance (NCHR) cantilevers (Nanotools USA LLC) (integrated spherical tips of radius 300 nm [±10%], nominal resonance frequency 330 kHz, and length 125 μm) were used.

Before indentation tests, the deflection sensitivity of the AFM cantilever was calibrated by engaging the probe on a clean glass slide. To calibrate the spring constant of the cantilever, the first free resonance peak of the cantilever was fitted to the equation of the simple harmonic oscillator so as to measure the power spectral density of the thermal noise fluctuation in air. The longitudinal and transverse elastic moduli (E_l_ and E_t_) of the samples were obtained from the force‐indentation depth curves through the Hertzian contact mechanics model, where $E=3F\left(1-\nu 2\right)/4\sqrt{R\delta 3}$ is the relation between the elastic modulus E and the applied indenting load F with ν being the Poisson's ratio of the sample, R the radius of the spherical probe, and δ the indentation depth. Any strain below the elastic limit was assumed infinitesimal, a condition satisfied with the use of an indentation depth below 50 nm that rules out the effect of the substrate as well as any inelastic behavior.^(^ ^24^, ^26^ ^)^ AFM data analysis was performed with the native JPK data processing software.

### Finite element analysis model creation

Micro‐CT scan data was segmented and a 3D rendered surfaces of the femurs were created using the ITK‐SNAP software (open source software, www.itk-snap.org)^(^ ^27^ ^)^; three‐dimensional solid geometries of femurs of the WT and conditional menin KO mice were then reconstructed by using SolidWorks software package (Dassault Systems SolidWorks Corporation, Waltham, MA, USA), and then discretized into volume mesh elements. Elastic moduli obtained from AFM indentation of KO and WT femur sections were assigned to cortical and trabecular bone models. Both KO and WT samples were assumed isotropic. This simplification did not yield noticeable diversions from the results of the orthotropic constitutive model.^(^ ^28^, ^29^ ^)^ The Hounsfield unit values (HU) were measured from the micro‐CT images and were used to assign the elastic properties to cortical and trabecular bones, as well as bone marrow. The mean values of longitudinal elastic moduli, El, of cortical and trabecular bones for KO and WT femurs were used, with a value for Poisson's ratio of 0.3.^(^ ^29^, ^30^ ^)^ Bone marrow was assumed as an incompressible isotropic material with elastic modulus E = 20 MPa with a Poisson's ratio of ν = 0.499.^(^ ^29^ ^)^

### Primary MSC isolation and culture

Six‐month‐old male and female KO and WT mice were euthanized, and their femurs and tibias were carefully dissected under sterile conditions. Epiphyses of each bone were cut and the marrow cells were harvested as previously described.^(^ ^17^ ^)^ The cells were cultured in a 5% CO_2_ incubator at 37°C in α‐minimal essential medium (αMEM, Invitrogen, Carlsbad, CA, USA; 12000‐022) supplemented with 10% FBS (Wisent, Saint‐Jean‐Baptiste, Canada; 080152), with 1% sodium pyruvate (Wisent; 600‐110‐EL), and 1% penicillin–streptomycin (Wisent; 450‐201‐EL) and 0.02 m sodium bicarbonate (Sigma; S5761‐500G). Medium was replaced every 2 to 3 days.

### Differentiation assays of MSC

Bone marrow cells were washed three times with sterile PBS to remove nonadherent cells. The adherent cell fraction was allowed to expand until 80% confluence was reached and then plated in 6‐well plates to determine alkaline phosphatase activity and calcium apposition. Cells were cultured in osteogenic medium containing 50 μg/ml L‐ascorbic acid (Sigma‐Aldrich) and 10 mM β‐glycerophosphate (Sigma‐Aldrich) for 14 days and then fixed with 100% ethanol.

To assess calcium deposition, cells were stained with 40 mM Alizarin Red‐S (Sigma‐Aldrich) at pH 4.2 and incubated for 30 minutes at room temperature with gentle shaking. Mineral accumulation was quantified as followed: Alizarin Red‐S was extracted by destaining with 10 mM HCl in 70% ethanol and the intensities quantified at 520 nm in 96‐well plates using a plate reader (Synergy H4 Hybrid Multi‐Mode Microplate Reader, BioTek, Winooski, VT, USA). ALP activity was determined by histochemical staining using NBT/BCIP tablets (Roche Diagnostics, Indianapolis, IN, USA). One ALP tablet was dissolved in 10 mL of distilled water and then added onto fixed cells followed by direct incubation at 37°C for 30 minutes in the dark. Stained wells were washed and air‐dried. To quantify ALP staining, 10× microscopic images were taken using the Evos XL Core light microscope (Life Technologies, Carlsbad, CA, USA) and relative staining was determined by calculating the mean gray area using the ImageJ software (NIH).

### Methyl thiazolyl tetrazolium (MTT) dye viability assay

Primary MSCs were seeded at a density of 2–3 × 10^4^ cells per well in 24‐well tissue cultures plate and cultured for 48 hours. MTT reagent (Roche Diagnostics) was then added, and incubation was continued for 4 hours. Formed crystals were eluted in 500 μL of DMSO and absorbance was read at 570 nm using a microplate reader (Synergy H4 Hybrid Multi‐Mode Microplate Reader, BioTek).

### Osteoblast/osteocyte‐osteoclast co‐cultures

Osteoblasts/osteocytes from long bones of 6‐ to 7‐month‐old KO and WT mice were isolated. Epiphyses were cut, marrow was flushed, and bones were transferred onto a Petri dish containing sterile PBS. Soft tissues were removed with a scalpel and the bones were chopped into small fragments. The bone fragments were incubated for 30 minutes at 37°C with shaking in 2 mg of collagenase II/ml αMEM (Bioshop, Burlington, Canada). The supernatant was discarded, and the procedure was repeated. Bones were then incubated for 30 minutes at 37°C with shaking in a 0.25% trypsin, 0.1% EDTA solution. The supernatant was discarded and replaced by fresh collagenase II solution for the fourth and final incubation step of 30 minutes at 37°C. Three subsequent washes with αMEM containing 10% FBS were done to remove all traces of Trypsin. The bone fragments were then transferred to T75 flasks and incubated in αMEM containing 10% FBS and 50 μg/mL l‐ascorbic acid without moving for 3 to 4 days. Adult mouse bone cells migrated from the bone chips after 48 hours, and after 5 to 6 days, the cells were counted and seeded (1 × 10^3^) in transwell insert devices for 24‐well plates (Thermo Fisher Scientific) with αMEM containing 10% FBS and ascorbic acid. Osteoblasts/osteocytes were allowed to reach 80% confluency (~5 days).

WT and KO osteoclast precursor cells were isolated from bone marrow according to Boraschi‐Diaz and colleagues.^(^ ^31^ ^)^ Briefly, long bones were cut in half, inserted into cut 1 mL pipette tips placed inside Eppendorf tubes, and centrifuged at 12,000*g*, three times for 30 seconds. The marrow was resuspended into αMEM by repeated pipetting up and down, transferred to a conical tube containing 10 mL of culture medium, and centrifuged at 1000*g* for 5 minutes. Red blood cells from the pellet were lysed by resuspending in red blood cell (RBC) lysis buffer (Sigma‐Aldrich). After incubation and washing, the final bone marrow cells pellet was resuspended in culture medium, plated in a 75 cm^2^ flask, and incubated overnight. The next day, nonadherent cells were pelleted by centrifuging the collected cell medium at 1000*g* for 5 minutes. The pellet was resuspended in 10 mL of culture medium and viable precursors were counted using Trypan blue (Sigma‐Aldrich). Precursors were then seeded (6 × 10^6^ per well) in 24‐well plates with αMEM (10% FBS) and allowed to settle for 12 to 16 hours. The transwell devices with grown osteoblasts/osteocytes were then inserted in each well containing osteoclast precursors and cultured in αMEM containing different concentrations (0, 5, 10, or 25 ng/mL) of RANKL neutralizing antibody (PA5‐47641, Thermo Fisher Scientific). Cells were further cultured for another 7 days, at which point, osteoclasts were observed and fixed with 10% paraformaldehyde in PBS, pH 7.4, for 10 minutes at room temperature and stained for tartrate‐resistant acid phosphatase (TRAP, Sigma; 387A). Osteoclast numbers were quantified by counting TRAP‐positive cells with three or more nuclei and intact membranes.^(^ ^32^ ^)^

### Statistical analysis

Results are expressed as mean ± standard deviation (SD). Statistical comparisons were made using the PRISM analysis software version 9.0 (GraphPad Software Inc., San Diego, CA, USA). One‐way analysis of variance was used to discriminate any differences among means of groups of data. Unpaired two‐tailed Student's *t* tests were used to assess significance; a *p* value <0.05 was taken to indicate a statistically significant difference.

## Results

### Disruption of the *Men1* gene with *PRX1‐Cre* is specific to tissues of mesenchymal origin

PCR amplification of tissue genomic DNA confirmed that *Prx1*‐mediated Cre recombination occurred in tissues of mesenchymal origin such as bone (femur, tibia, calvaria), white adipose tissue (WAT), cartilage, and muscle but not in kidney of the KO *Prx1‐Cre*; *Men1* ^*f/f*^, mice (Fig. 1*B*). No recombination was observed in *Men1* ^*f/f*^ (WT) mice.

### Deletion of menin in mesenchymal stem cells results in altered bone morphology and reduced muscle strength

Gene expression levels of menin were measured by qRT‐PCR and were found to be reduced by about 60% in femurs and tibias of KO mice; in contrast, *MEN1* expression was not altered in kidney (Fig. 1*C*). Menin mRNA levels were also reduced by ~65% in cartilage, muscle, and white adipose tissue (Fig. 1*C*). Conditional inactivation of *MEN1* in mouse MSCs led to a decrease in overall size of KO mice relative to control WT mice (Fig. 1*D*). By radiography, femur epicondyles were shown to be morphologically altered, particularly at the level of the greater and lesser trochanters (Fig. 1*E*), and quantitative assessment of femur length at 6 months, taken as the diagonal distance between the femoral head and the lateral condyle, demonstrated a statistically significant reduction in femur length in the KO mice relative to WT (Fig. 1*E*). The weight of KO mice was significantly reduced postnatally through 21 weeks of age in both males and females (Fig. 1*F*), and Echo‐MRI analysis of KO males at 12 months of age demonstrated a significant 11% reduction in lean mass weight relative to WT (Fig. 1*G*). To determine whether changes observed in body metabolism and in lean mass content could affect skeletal muscle strength, a forelimb grip strength test was performed (Fig 1*H*). Individual values obtained from the grip strength test were normalized to total body weight of each mouse. Six‐month‐old KO male and female mice demonstrated a significant decrease of ~30% in forelimb grip strength in comparison to control WT animals. The lean mass and grip strength phenotypes observed were not significantly different between sexes.

### Deletion of menin in mesenchymal stem cells results in reduced bone mineral density but intact cartilaginous growth plates

**Fig. 2:**
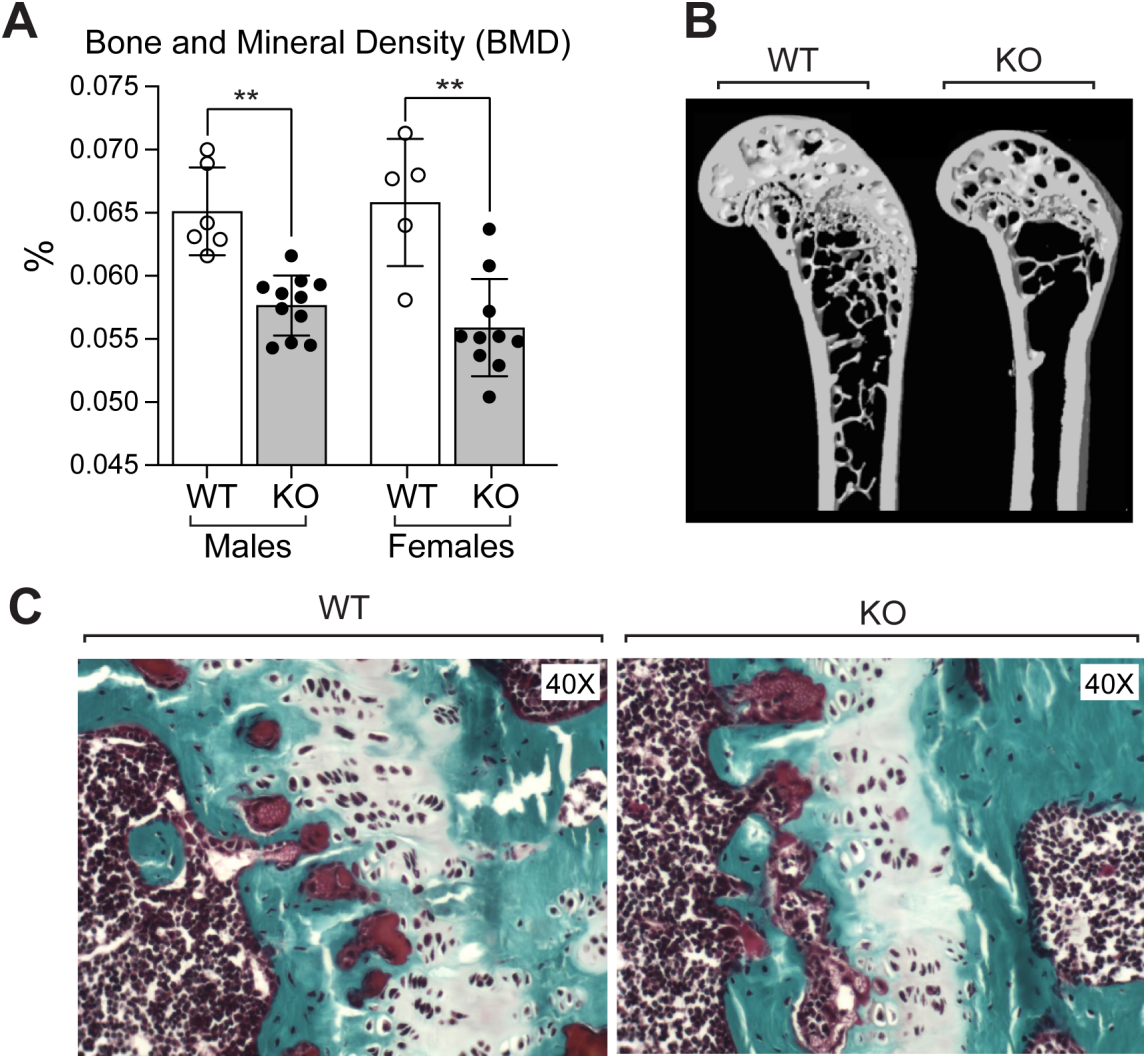
(*A*) Bone mineral density (BMD) assessed by dual‐energy X‐ray aborptiomtry (DXA) analysis in femurs of male and female mice at 7 months of age. (*B*) Reconstructed distal femur models generated from micro‐CT scans of 9‐month‐old WT and KO male mice. Data are presented as mean ± SD; statistical significance indicated on plots: **p < 0.01. (*C*) Histology of femoral growth plates of WT and KO mice. In each case, representative photomicrographs of the growth plate are shown stained by trichrome stain. Magnification ×40.

BMD, determined by DXA, was shown to be significantly decreased in KO mice by approximately 11% in males and approximately 13% in females (Fig. 2*A*). Qualitatively, reconstructed distal femur models generated from micro‐CT scans showed reductions in trabecular bone in femurs of KO mice (Fig. 2*B*). Nevertheless, cartilaginous growth plates of WT and KO mice were not different and both showed similar intact growth plates (Fig. 2*C*), indicating that chondrocyte alteration did not contribute substantially to observed changes in postnatal bone mass acquisition. The phenotypes observed were similar in both sexes.

### Quantitative micro‐CT demonstrates altered trabecular bone in knockout mice

**Fig. 3:**
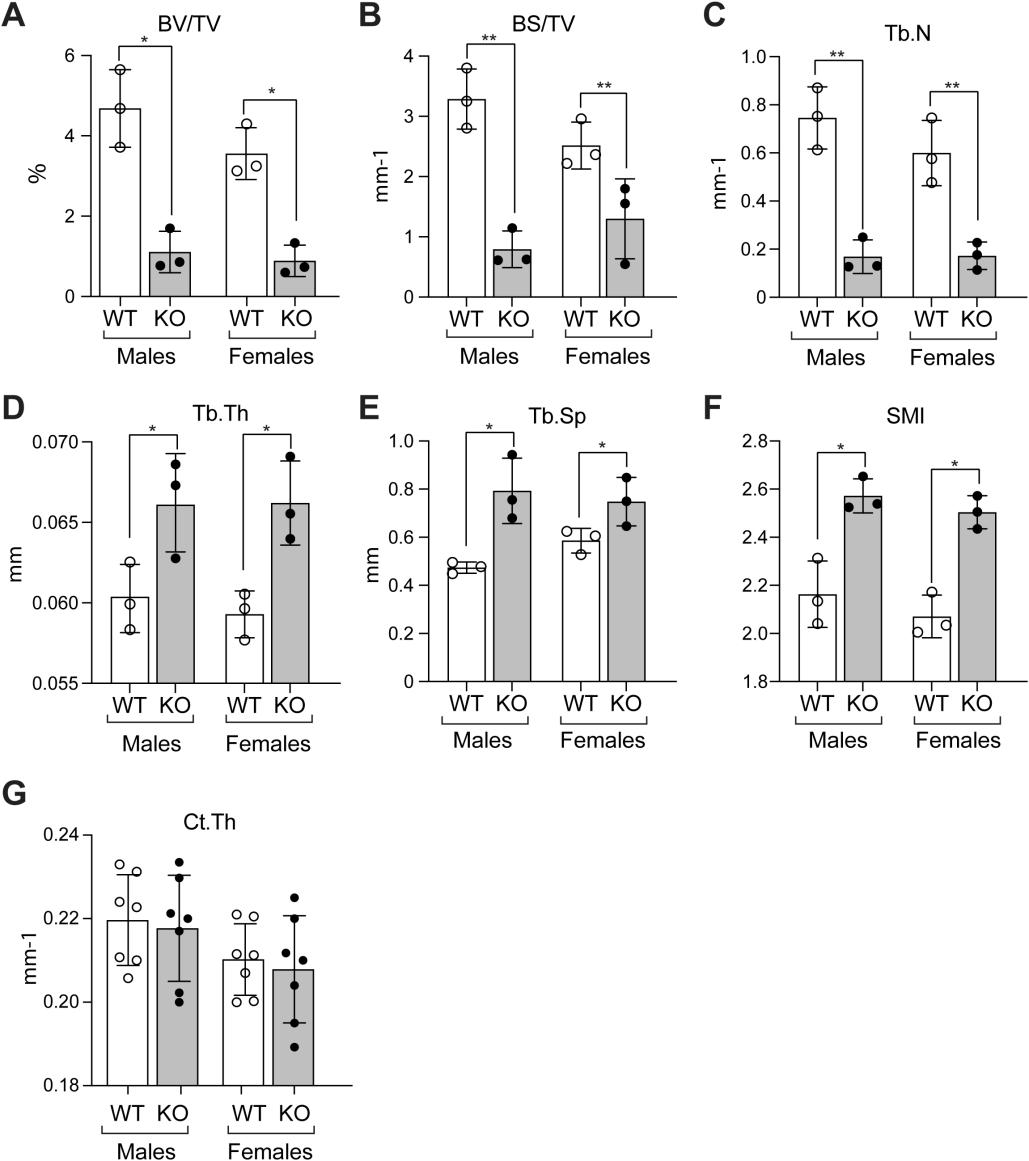
Quantitative analysis of data from three‐dimensional micro‐computed tomography (micro‐CT) of distal femurs of 6‐ to 7‐month‐old male and female WT and KO mice. (*A*) Bone volume/total volume (BV/TV), (*B*) bone surface/total volume (BS/TV), (*C*) trabecular number (Tb.N), (*D*) trabecular thickness (Tb.Th, (*E*) trabecular separation (Tb.Sp), (*F*) structure model index (SMI), and (*G*) cortical thickness (Ct.Th). Data are presented as the mean ± SD of three (*A–F*) and seven (*G*) determinations. Statistical significance indicated on plots: *p < 0.05, **p < 0.01.

Marked reductions were observed In both male and female KO mice, relative to WT mice, in trabecular bone volume (BV/TV) (75% in both males and females, Fig 3*A*), trabecular bone surface (BS/TV) (75% in males and 52% in females, Fig 3*B*), and trabecular number (Tb.N) (77% in males and 72% in females, Fig. 3*C*); in contrast, increases were observed in both male and female KO mice in trabecular thickness (Tb.Th) (10% in both males and females, Fig. 3*D*) and trabecular separation (Tb.Sp) (65% increase in males and 72% increase in females, Fig. 3*E*) relative to WT mice. The increase in structure model index (SMI; 20% increase in both males and females, Fig. 3*F*) suggested abnormal trabecular structure with potentially compromised mechanical integrity in KO mice. Cortical thickness (Ct.Th) (Fig. 3*G*) was not altered. These findings suggest that menin functions critically at early stages of the osteoblast lineage to regulate trabecular bone development and maintenance in vivo.

**Fig. 4:**
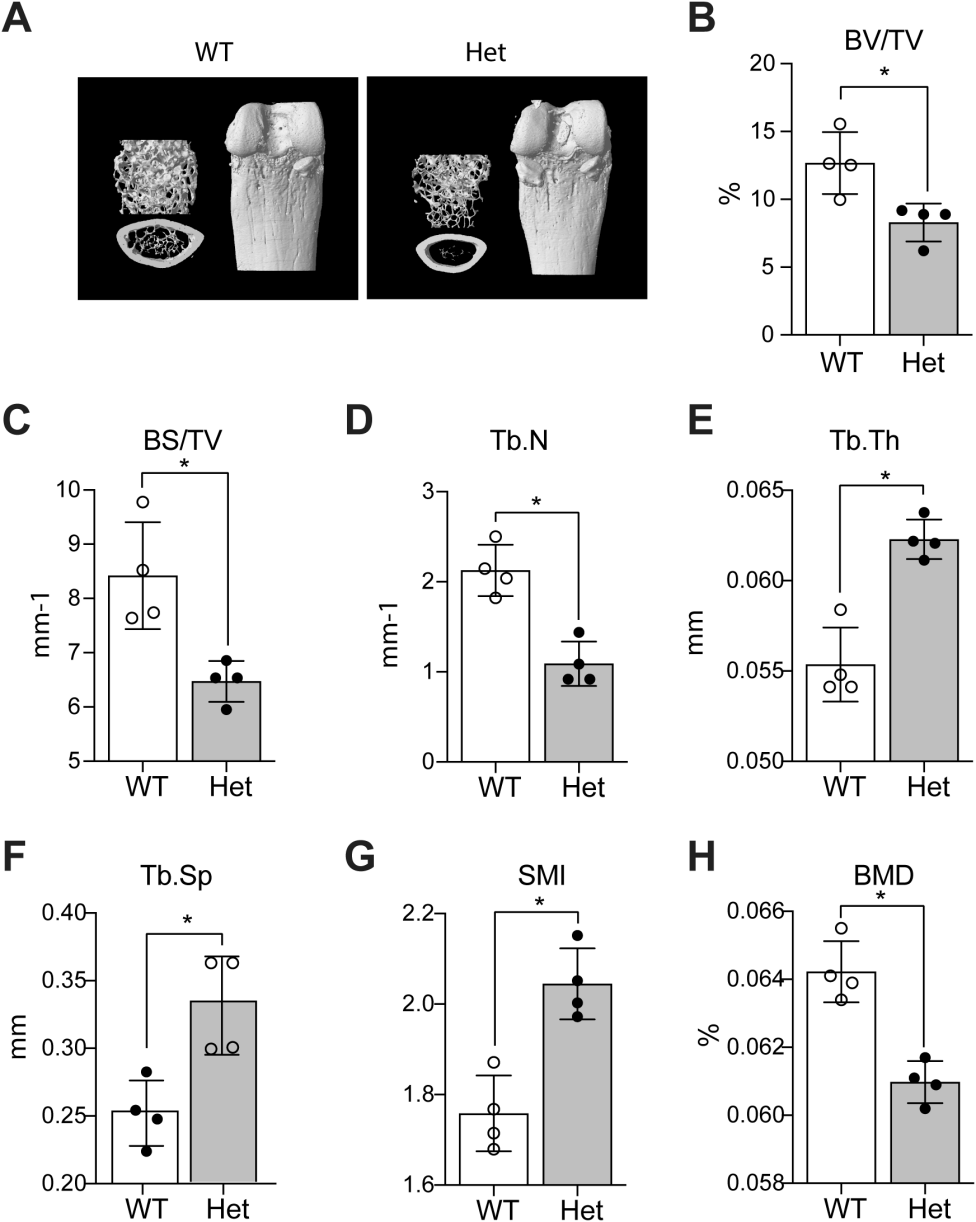
Qualitative and quantitative analysis of distal femurs of WT and heterozygous (Het) 6‐ to 7‐month‐old male mice. (*A*) Representative longitudinal sections (upper left) and cross sections (lower left) of 3D reconstructed distal ends of femora by micro‐CT and representative vertical view of intact distal ends of femurs (right). (*B*–*F*) Quantitative analysis of data from three‐dimensional micro‐computed tomography of distal femurs of WT and heterozygous mice showing (*B*) bone volume/total volume (BV/TV), (*C*) bone surface/total volume (BS/TV), (*D*) trabecular number (Tb.N), (*E*) trabecular thickness (Tb.Th), (*F*) trabecular separation (Tb.Sp), (*G*) structure model index (SMI), and (*H*) bone mineral density (BMD) assessed by DXA analysis of femurs. Data are presented as the mean ± SD of four determinations. Statistical significance indicated on plots: *p < 0.05.

Less marked but significant reductions in trabecular bone were also observed both qualitatively (Fig. 4*A*) and quantitatively (BV/TV reduction of 35%, BS/TV reduction of 23%, Tb.N reduction of 50%, Fig. 4*B–D*) in male mice with heterozygous menin deletion. Significant increases were also found for Tb.Th (13%), Tb.Sp (25%), and SMI (15%) (Fig. 4*E–G*). BMD was significantly reduced by 5.1% (Fig. 4*G*).

### Menin inactivation in mesenchymal stem cells results in unaltered osteoblast numbers and bone formation but increased osteoclastogenesis in vivo and in vitro

**Fig. 5:**
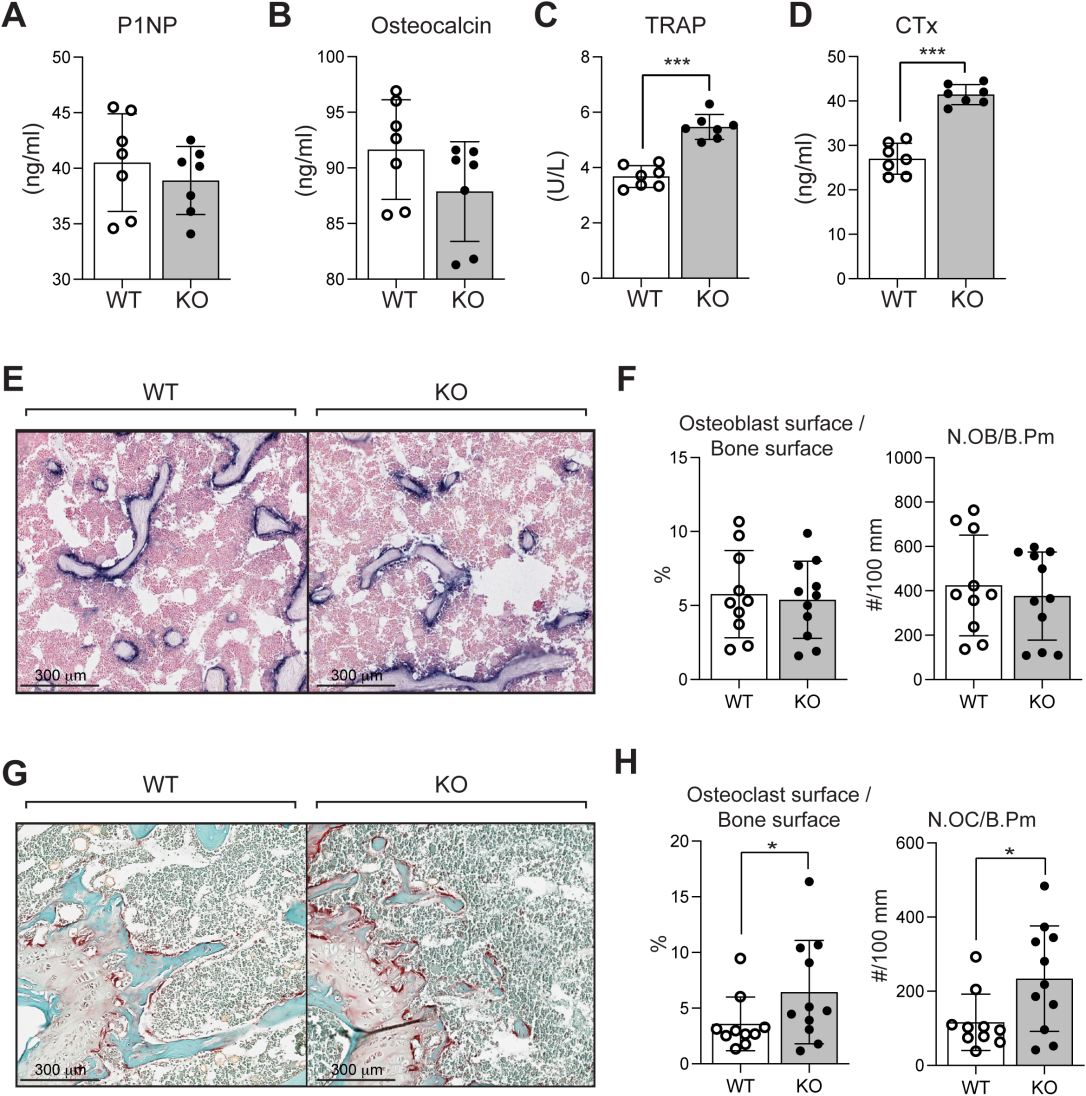
(*A–D*) Elisa quantitation of serum levels of bone markers in 6‐month‐old WT and KO male mice: (*A*) procollagen 1 intact N‐terminal propeptide (P1NP), (*B*) osteocalcin, (*C*) tartrate‐resistant acid phosphatase (TRAP), and (*D*) C‐telopeptide degradation products of type 1 collagen (CTX‐1). Each value is the mean ± SD of seven determinations. (*E–H*) Histomorphometric assessment of osteoblasts and osteoclasts: (*E*) representative paraffin sections of femurs from 6‐month‐old WT and KO male mice stained with alkaline phosphatase for assessment of osteoblasts numbers. Scale bar = 300 μm quantitation of osteoblasts (*F*), including osteoblast surface relative to bone surface and osteoblast numbers normalized to bone perimeter (N.OB/b.pm). (*G*) Representative paraffin sections of femurs from 6‐month‐old WT and KO male mice stained with TRAP for assessment of osteoclast numbers. Scale bar = 300 μm. Each value is the mean ± SD of 10 and 11 determinations in WT and KO mice, respectively. Quantitation of osteoclasts (*H*), including osteoclast surface relative to bone surface and osteoclast numbers normalized to bone perimeter (N.OC/b.pm). Each value is the mean ± SD of 10 and 11 determinations in WT and KO mice, respectively. Statistical significance indicated on plots: *p < 0.05, ***p < 0.001.

Serum bone marker determinations showed normal amino‐terminal propeptide of type 1 procollagen (P1NP) and osteocalcin levels (Fig. 5*A*, *B*) but a ~50% increase in both tartrate‐resistant acid phosphatase (TRAP) and C‐telopeptide of type I collagen (CTX‐1) levels (Fig. 5*C*, *D*) in KO mice, consistent with increased osteoclastic activity. Decalcified and paraffin‐embedded femur sections from 6‐ to 7‐month‐old WT and KO mice were stained with ALP (Fig. 5*E*) and TRAP (Fig. 5*G*) for the assessment of osteoblast numbers and osteoclast numbers, respectively. Osteoblast numbers were unchanged in KO mice relative to WT littermates (Fig. 5*F*); however, significant increases in the number of osteoclasts per bone perimeter were observed (100% increase, Fig. 5*H*).

**Fig. 6:**
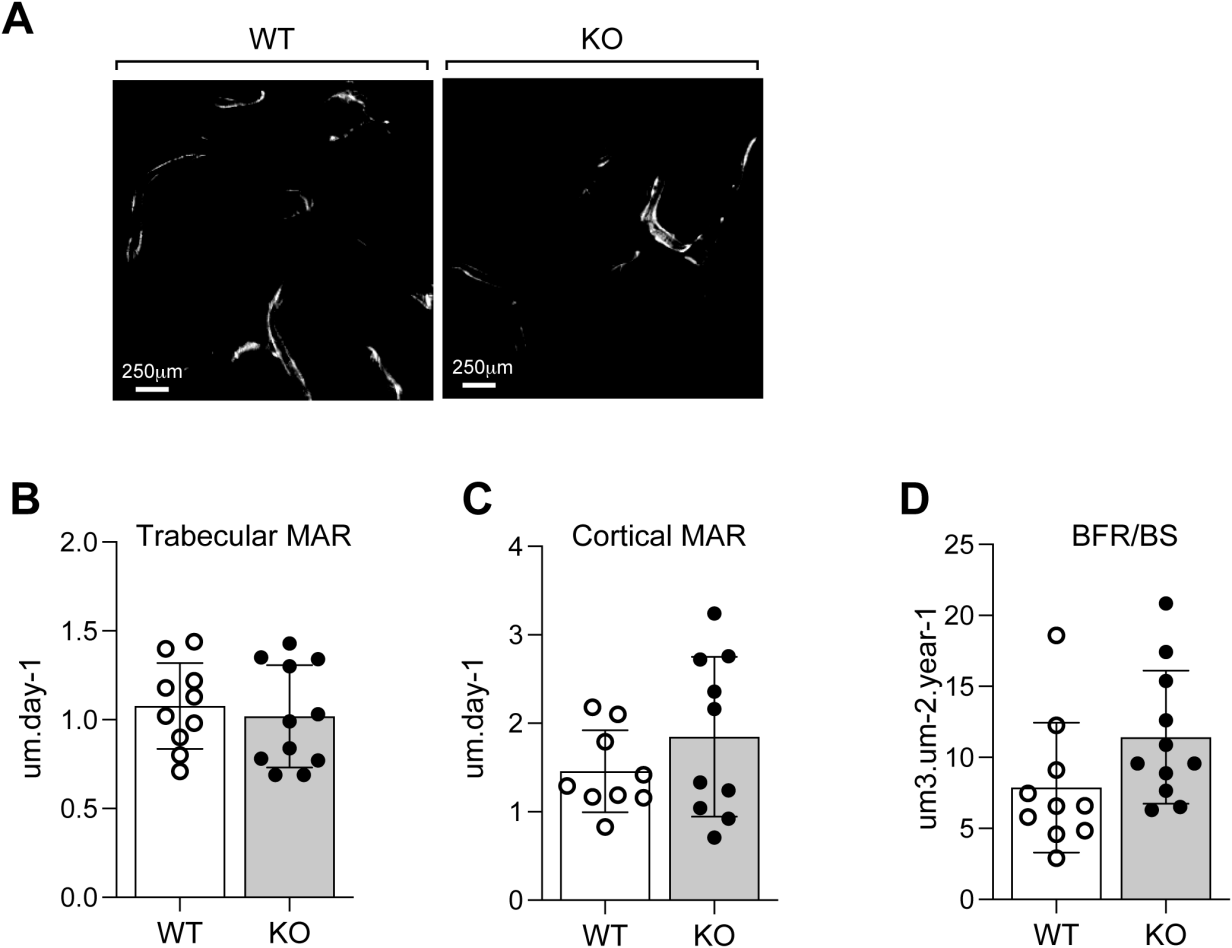
Dynamic histomorphometric analysis. (*A*) Representative unstained polymethylmethacrylate (PMMA)‐embedded femur sections of 6‐month‐old WT and KO females examined under fluorescent light to visualize double‐calcein labeling; scale bar = 250 μm. Indices of bone formation calculated were: (*B*) trabecular mineral apposition rate (MAR), (*C*) cortical MAR, and (*D*) the bone formation rate/bone surface (BFR/BS). Each value is the mean ± SD of 10 (WT) and 11 (KO) determinations.

Dynamic histomorphometric parameters were assessed by double calcein labeling in 6‐ to 7‐month‐old female mice (Fig. 6*A*). Trabecular (Fig. 6*B*) and cortical (Fig. 6*C*) mineral apposition rate (MAR) and trabecular bone formation rate/bone surface (BFR/BS) (Fig. 6*D*) were unaltered in KO mice relative to WT. Results in male mice were similar (data not shown). These findings indicate that the significant alterations in bone microscopic and macroscopic parameters observed in KO mice appear to result from an increase in bone resorption, rather than a decrease in formation.

**Fig. 7:**
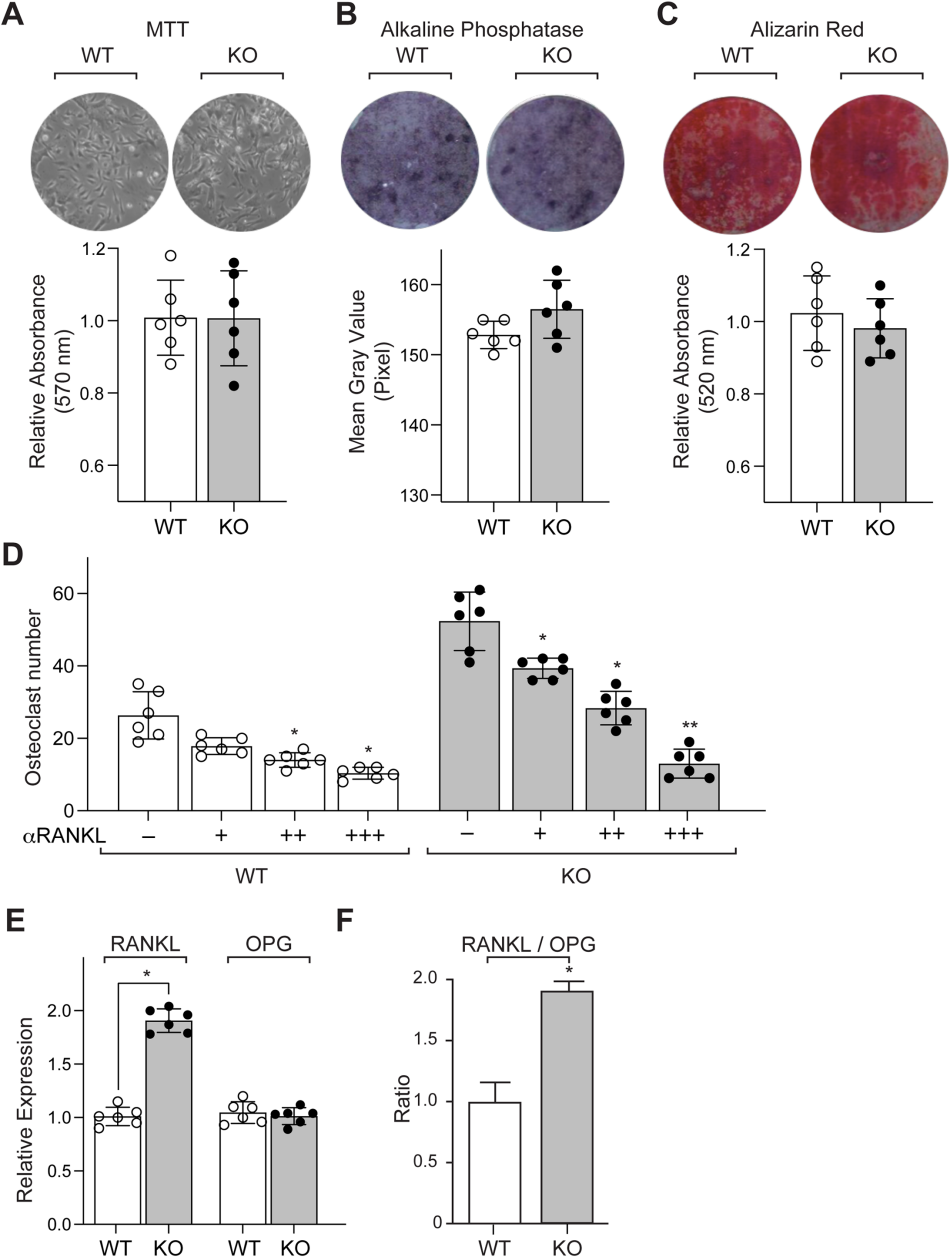
Mesemchymal stem cells (MSCs) isolated from bone marrow of femurs of 6‐month‐old WT and KO mice were cultured in vitro for 7 to 10 days and (*A*) stained for viability using methyl thiazolyl tetrazolium (MTT) dye. Absorbance was read at 570 nm and normalized to the 600‐nm value (*n* = 6). MSCs were cultured for 14 days in osteogenic medium containing β‐glycerophosphate and l‐ascorbic acid (*n* = 6) and stained for (*B*) alkaline phosphatase (ALP) for differentiation and (*C*) Alizarin Red‐S for mineralization. (*D*) Trap‐positive osteoclast numbers from co‐cultures of osteoblasts/osteocytes and bone marrow osteoclast precursors in the presence of increasing concentrations, ie, 0 (−), 5 (+), 10 (++), or 25 (+++) ng/mL of RANKL neutralizing antibody (αRANKL). Each value represents the mean ± SD of six replicates. (*E*) RT‐qPCR assessment (*n* = 4) of gene expression profiles of RANKL and OPG from femurs of WT and KO mice, normalized to GAPDH expression, and (*F*) the ratios of RANKL/OPG. Each value represents the mean ± SD. Statistical significance indicated on plots: *p < 0.05, **p < 0.01.

To further assess potential differences in osteoblast function responsible for the osteopenic phenotype observed in KO mice, MSCs were isolated and studied in vitro. MTT assay revealed no significant differences in proliferative capabilities of MSC cultures from KO mice relative to WT at 48 hours after first passage (Fig. 7*A*). When MSC cultures were treated for 14 days with osteoblast differentiation media, no difference was observed in differentiation and mineralization capabilities as illustrated by alkaline phosphatase (ALP) (Fig. 7*B*) and Alizarin Red (AR) staining (Fig. 7*C*), respectively. Taken together, these findings demonstrate that differentiation and mineralization of mature osteoblasts are unaltered in vitro after the deletion of menin in the mesenchymal stem cell. In contrast, the capacity to generate osteoclasts in bone marrow cultures in vitro was significantly increased in KO mice and a blocking antibody to receptor activator of NF‐κB ligand (RANKL) reduced the increased osteoclast numbers observed (Fig. 7*D*).

To further evaluate the molecular basis of the increase in osteoclast numbers, RANKL and osteoprotegerin (OPG) mRNA levels were examined in long bones of WT and KO mice (Fig. 7*E*, *F*). Quantitative real‐time PCR conducted on total mRNA isolated from the femurs of 6‐ to 7‐month‐old animals demonstrated significant increases of RANKL mRNA levels in KO relative to controls (approximately 100%, Fig. 7*E*). OPG mRNA levels were not significantly elevated (Fig. 7*E*) and RANKL/OPG levels were increased (approximately 100% increase, Fig. 7*F*).

### Deletion of menin in mesenchymal stem cells impairs femur trabecular and cortical microstructure, mechanics, and nano‐dynamic elastic properties

**Fig. 8:**
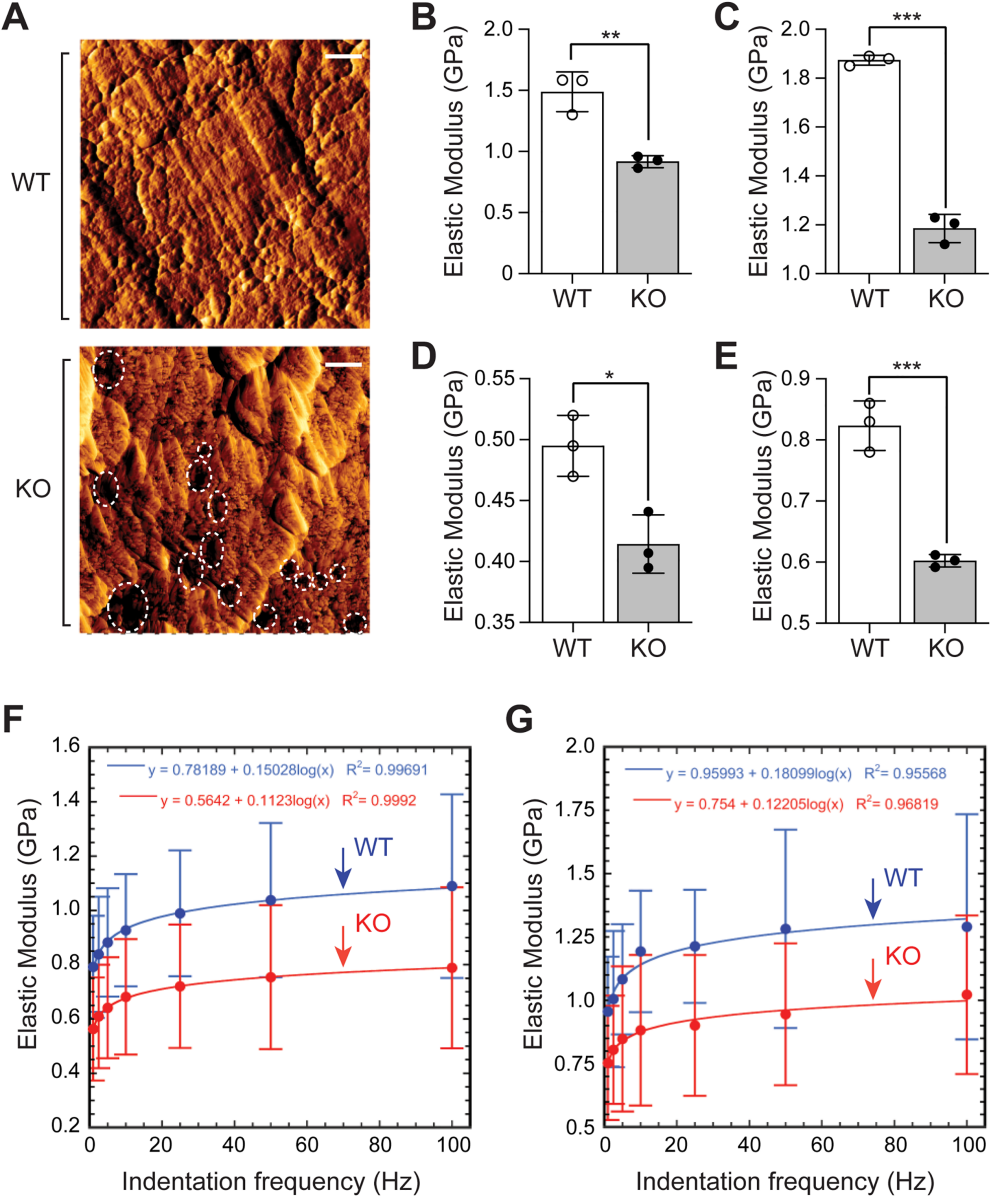
(*A*) Error signal AFM contact mode images of areas within the cortex in WT and KO samples. Pores within the cortex are shown by dashed circles. Scale bar = 500 nm. The structure of cortex in KO samples is found to be more porous than that in WT samples. Comparison of cortical bone (*B*) transverse elastic moduli (Et) and (*C*) longitudinal elastic moduli (El) of femurs from WT and KO mice. (*D*, *E*) Counterpart comparison for the elastic moduli of trabecular bone in WT and KO samples. Each value represents the mean ± SD (*n* = 3). Comparison of elastic moduli of WT (blue) and KO (red) elastic moduli obtained via AFM indentation. (*F*) Longitudinal elastic modulus El of cortical bone in WT and KO mice femurs at selected values of indentation frequency from 1 to 100 Hz plotted with a logarithmic fit. (*G*) El of trabecular bone in WT and KO mice at frequencies in 1 to 100 Hz plotted with a logarithmic fit. Results are expressed in gigapascals (GPa). Statistical significance indicated on plots: *p < 0.05, **p < 0.01, ***p < 0.001.

Atomic force microscope (AFM) indentation was conducted on cortical and trabecular bone samples using both transverse and longitudinal sections, as previously described.^(^ ^24^ ^)^ Qualitative microscopic geometric differences in cortical bone structure were observed in femur sections obtained from KO mice, most notably, a more porous architecture that is less monolithic than that observed in WT controls (Fig. 8*A*). The anisotropy ratios of femurs (ie, the ratios of the average values of the longitudinal elastic modulus El, over the transverse elastic modulus Et) for KO samples ranged from 1.3 to 1.5, in contrast to values of 1.2 to 2 for WT mice. The mean values of cortical bone elastic moduli in the transverse (Et) (Fig. 8*B*) and longitudinal (El) (Fig. 8*C*) directions were found to be 51% and 43% larger in WT femur samples, respectively, relative to their corresponding KO littermates. Similar significant differences were observed for trabecular bone; mean Et and El values in WT trabecular samples were found to be 25% and 33% greater than what was observed in KO, respectively (Fig. 8*D*, *E*). Statistically significant differences were found between the mean values of Et and El in WT cortical bone.

To compare the dynamic elastic behavior of WT and KO samples, additional indentation tests were performed at given frequencies from 1 to 100 Hz.^(^ ^24^, ^26^ ^)^ For each sample examined, within the cortical (Fig. 8*F*) and trabecular (Fig. 8*G*) bone compartments indented, a logarithmic fit of the correlation between the longitudinal elastic modulus El and the indentation frequency was established, as presented in Fig. 6*F*, *G*. Depending on the frequency, the mean value of El in WT cortical bone was found to be 15% to 38% greater than in KO counterparts (Fig. 8*F*); the mean El value in WT trabecular samples was 23% to 35% greater in WT than in KO mice (Fig. 8*G*).

Taken together, these findings indicate that deletion of menin early in the osteoblast lineage impairs femur trabecular and cortical microarchitecture and compromises its mechanical and dynamic elastic properties at the nanoscale.

### Computational and experimental analyses demonstrate significantly reduced mechanical alterations in menin‐deficient femurs

**Fig. 9:**
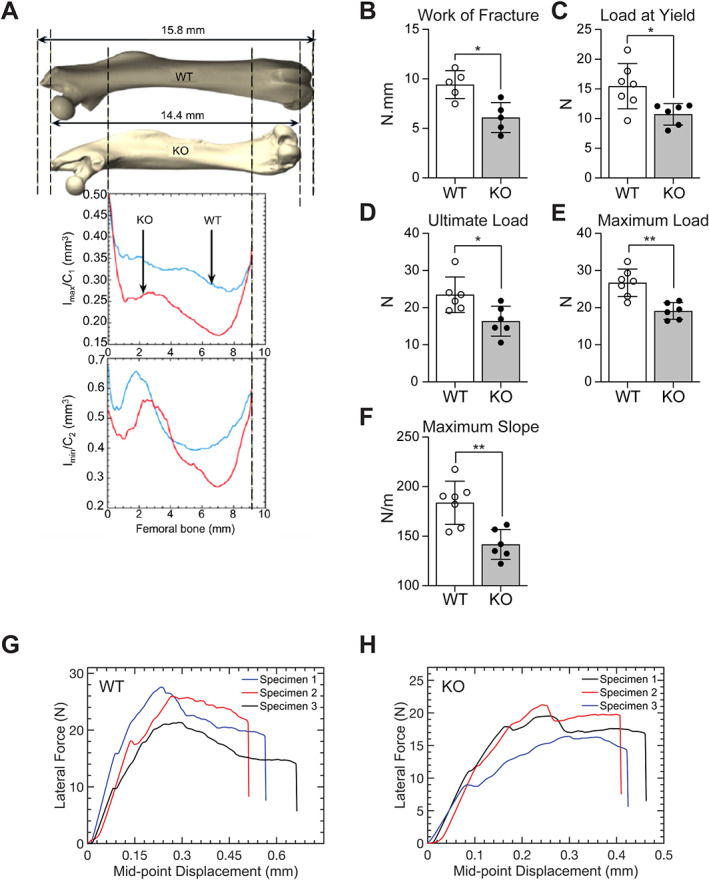
(*A*) Geometric bending parameters of cortical bone in wild‐type (WT; blue) and knockout (KO; red) femur samples. Coefficients Imax/C1 and Imin/C2 were obtained in three pairs of KO and WT mice at 9 months of age. (*B–H*) Results, for WT and KO femurs, of destructive three‐point bending experiments. Analysis of (*B*) the work of fracture (ie, the area under the force‐deflection curves), (*C*) the load at the yield point, (*D*) the load at ultimate failure (ultimate load), (*E*) the maximum load, and (*F*) the maximum slope. Each value is the mean ± SD of five to seven determinations. (*G*, *H*) Force‐displacement curves for three specimens of WT and KO femora. N = newtons. Statistical significance indicated on plots: *p < 0.05, **p < 0.01.

Fig. 9*A* shows the geometric portion of flexural rigidities (ie, the capacity of the beams to support lateral forces) for femurs of WT and KO mice. Bending coefficients Imax/C1 and Imin/C2 (with Imax and Imin being the principal second moments of inertia of the femur lateral cross section, and C1 and C2 the farthest distances of material elements in each cross section from the two principal bending axes of that cross section) were obtained using the micro‐CT data of three pairs of KO and WT mice at 9 months of age. As demonstrated in Fig. 9*A*, geometric bending stiffness was significantly reduced along the femoral shaft by up to 50% at the level of the diaphysis in femurs of KO mice. This geometric feature, together with the longitudinal and transverse elastic moduli, E_l_ and E_t_ (obtained through AFM indentation testing) describes the overall bending stiffness of the femoral bone as a comparative tool to highlight the differences in bending capacities of WT and KO samples.

Three‐point bending tests were then conducted to assess differences in macroscopic elastic properties at the level of the mid‐diaphysis. The work of fracture (Fig. 9*B*), load at the yield point (Fig. 9*C*), and load at ultimate failure (Fig. 9*D*) were all significantly reduced in KO femurs relative to WT. Thus, the work of fracture in WT samples was 40% larger than the corresponding value in KO samples, the load at yield in WT samples was 60% larger than that in KO ones, and the ultimate load in WT samples was 60% larger than that in KO ones. The average value of the maximum load supported by WT samples was 50% greater than that measured in KO mice, at 27 N and 18 N, respectively (Fig. 9*E*). Young's modulus, taken as the mean value of the maximum slope of the force‐displacement curve, was measured as 180 N/m in WT femurs, a value 30% greater than that in KO mice (140 N/m) (Fig. 9*F*). The average value of the maximum strain up to failure supported by WT samples was 0.57, which was found to be 29.5% greater than that measured in KO mice with maximum strain of 0.44 (Fig. 9*G*, *H*).

**Fig. 10:**
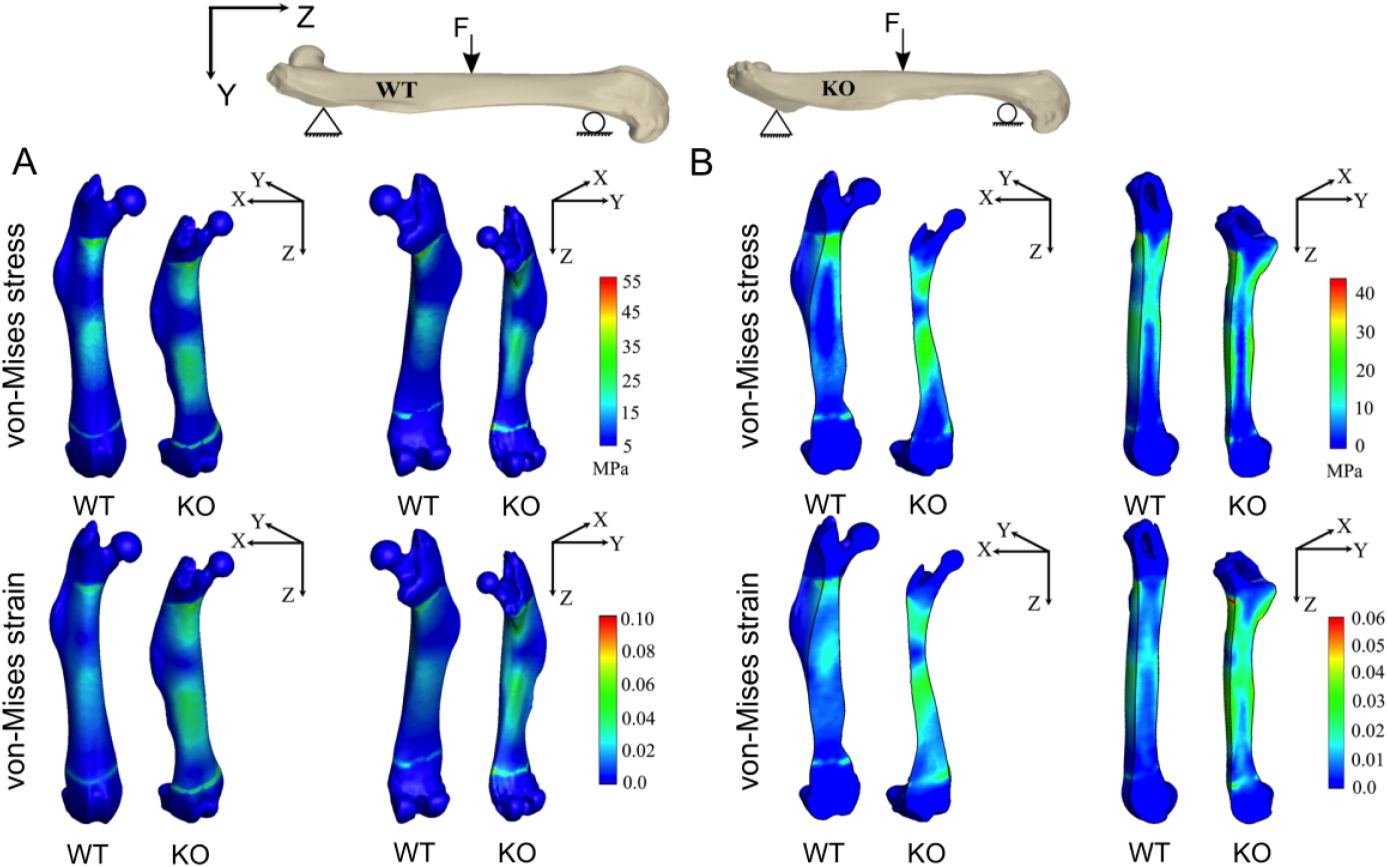
Results from finite element (FE) analysis of elastic three‐point bending for wild‐type (WT) and knockout (KO) samples. Shown are the distributions of von Mises stress and von Mises strain in the WT (left) and KO (right) femurs as a result of elastic three‐point bending. (*A*) Full view from the anterior (left images) and posterior (right images) of the femurs. (*B*) Frontal cross‐sectional view (left images) in X–Z plane, and sagittal cross‐sectional view (right images) in Y–Z plane, respectively. The color spectrum highlights the differences in each case.

Elastic 3‐point bending tests were then replicated using a computational approach as per the American Society for Testing and Materials (ASTM) standards for mechanical testing; femurs were modeled as simply supported beams with a 5 N concentrated load applied at the midspan. Femurs were then arranged such that condyles were positioned inferiorly to replicate the 3PBT experimental design. Simulation outcomes were cast into performance metrics to capture changes in the mechanical behavior in response to the applied loads for given boundary conditions. In particular, distributions of von Mises stress and von Mises strains were obtained in three pairs of WT and KO samples, with a representative pair presented in Fig. 10, for the anterior, posterior, transverse, and sagittal views. Quantitatively, KO femurs showed a 44% higher von Mises stresses than control samples, consistent with lower flexural stiffness in response to bending. In addition, KO femurs exhibited 75% larger von Mises strains relative to controls.

Taken together, these findings demonstrate that for the prescribed boundary conditions simulated and experimentally evaluated, deletion of menin in mesenchymal stem cells significantly reduced global bending stiffness and compromised macroscopic femur biomechanical strength.

## Discussion

Expression of the homeobox gene *Prx1* is restricted to the mesoderm during embryonic development^(^ ^33^ ^)^ and can regulate differentiation of mesenchymal precursors. Using the *Prx1* cre driver, conditional deletion of *Men1* in mesenchymal stem cells demonstrated significant reductions in overall weight throughout life, as well as a reduction in bone mineral density and femur length compared with WT littermates. Furthermore, lean weight and grip strength were reduced. This may reflect *Prx1‐Cre* expression in muscle as well as bone in keeping with the mesenchymal origin of both tissues (Fig. 1*C*) and the consequent deletion of menin in both tissues. Nevertheless, mice with conditional *Men1* knockout in mesenchymal stem cells showed intact epiphyseal growth plates (Fig. 2*C*). Micro‐CT imaging showed a decrease in trabecular bone volume, altered trabecular structure, and increase in trabecular separation but unaltered cortical bone thickness. Similar, although less severe, changes were observed in heterozygous mice with menin deletion. Dynamic histomorphometry demonstrated unaltered in vivo bone formation metrics and histologically, the number of osteoblasts was unchanged. In vitro proliferation of bone marrow stem cells isolated from KO mice, and differentiation and mineralizing capabilities of osteoblasts were unaltered compared with WT. Nevertheless, there was a marked increase in osteoclast number, consistent with our in vivo findings of increased circulating TRAP and CTX‐1 levels and increased RANKL/OPG mRNA profiles in bone. These findings supported increased osteoclastogenesis and bone resorption signaled by osteoblasts in reducing bone mass in menin‐deficient mice.

Increasingly, it has been recognized that measurement of BMD alone may not accurately demonstrate bone fragility and predisposition to fracture, and additional approaches to complement BMD measurements in osteoporosis have been reported.^(^ ^34^ ^)^ We, therefore, provided a detailed analysis of the biomechanical properties of bone caused by deletion of menin early in the osteoblast lineage. Thus, the mechanical behavior of bone is strongly dependent on its complex organization and hierarchical structure.^(^ ^35^, ^36^ ^)^ At the micro scale, the local response of bone to mechanical deformation is mainly a function of its material properties, namely, the spatial organization of hydroxyapatite crystals and collagen.^(^ ^37^ ^)^ At the macroscopic scale, its microstructural material properties and macro‐geometry regulate its mechanical behavior.^(^ ^38^ ^)^ Given the significant skeletal phenotype observed, we examined femur biomechanics and found that elastic properties were decreased at the femoral mid‐diaphysis with significant reductions in stiffness, maximum load, the work of fracture, the ultimate load, and the load at yield point.

Macroscopic testing such as three‐point bending tests cannot elucidate the microscopic differences in bone resulting from a gene knockout,^(^ ^25^, ^27^, ^35^ ^)^ nor does it provide information on the internal distribution of stresses and strains within the bone tissue in response to given loading conditions.^(^ ^27^, ^35^ ^)^

Differences in elastic properties along both the longitudinal and transverse axes were measured in both KO and WT femur samples. This confirmed a high level of local anisotropy in both trabecular and cortical bone tissues.^(^ ^24^ ^)^ Compared with the elastic moduli of cortical and trabecular bones in the transverse plane, a 20% to 50% increase was observed in the longitudinal direction in both KO and WT strains. The transverse section of femoral bone showed higher longitudinal elastic moduli compared with the transverse elastic moduli in the longitudinal section in both WT and KO samples. Furthermore, elastic moduli were up to 70% greater for cortical bone than for trabecular bone, with the lowest elastic modulus being that of trabecular bone in the transverse direction in both KO and WT bones. Also, in both KO and WT samples, El was found to be greater than Et at the same anatomical location within a given femur. Elastic moduli were greater at higher indentation frequencies in both KO and WT mice and in both cortical and trabecular bone compartments. For the same indentation depth, δ (ie, smaller than 50 nm), the indenting force, F, increased with the loading rate, thus demonstrating the dependence of bone stiffness on loading rate. This variation can be attributed to the proteinaceous nature of collagen fibrils at the molecular scale,^(26^ ^)^ and the direct correlation between elastic modulus and frequency is a result of the viscoelastic nature of bone.^(^ ^39^ ^)^ However, Et and El values of cortical bone were found to be significantly smaller in KO femur samples compared with their corresponding values in WT samples. Concomitantly, Et and Et values in KO trabeculae were observed to be significantly smaller than their counterparts in WT trabeculae. The sensitivity of this technique is thus paramount for an adequate assessment of the biomechanical phenotype of bone murine models such as the current one.

AFM experiments confirmed significant reductions in trabecular and cortical bone stiffness along both longitudinal and transverse planes in femurs of KO mice relative to controls. Computational analysis and simulation of the three‐point bending tests, using elastic properties obtained via AFM indentation, corroborated the low flexural stiffness observed experimentally. AFM proved valuable not only in demonstrating differences in nanoscale mechanical properties of KO femurs but also in documenting and establishing differences in bone mechanics within the same femur sample at different locations and orientations.^(^ ^24^ ^)^ The experimental, computational, and simulation biomechanical analyses adopted in this study thus unveil properties and performance metrics that would otherwise be dismissed with classical testing at the component level. This multiscale approach can therefore be used and applied in future studies as part of the phenotypic characterization of other murine models of bone development and bone physiology, providing useful insight pertaining to the integrity of bone and its behavior and extending the clinical utility of what BMD, X‐ray, and micro‐CT scan data can presently offer.

The tumor suppressor menin can impact several downstream signaling sytems, eg, menin can directly interact with Smad3 protein, and inactivation of menin blocks TGF‐β and activin signaling in anterior pituitary cells, which can antagonize their growth‐inhibiting properties.^(^ ^7^, ^8^ ^)^ Mediated by the Smads and the transcription factor, Pit‐1, menin can also cause activin‐induced inhibition of prolactin expression.^(^ ^8^ ^)^ In parathyroid cells, menin reduction can lead to loss of TGF‐β inhibition of parathyroid cell proliferation and parathyroid hormone (PTH) secretion.^(^ ^12^ ^)^ Menin was shown to regulate canonical Wnt/β‐catenin, and overexpression of menin reduced nuclear accumulation of β‐catenin and suppressed its transcriptional activity. Menin may also control endocrine cell proliferation, apoptosis, and DNA repair, at least in part by regulating histone modifications and chromatin structure, leading to altered gene transcription.^(^ ^40^ ^)^ Menin also epigenetically represses Hedgehog signaling, a proproliferative and oncogenic pathway.^(^ ^41^ ^)^

Although menin can suppress tumorigenesis in the endocrine lineage, it can also promote oncogenic function in other tissues, such as breast.^(^ ^42^ ^)^ In the hematopoietic lineage, menin serves as a molecular adaptor that physically links the mixed‐lineage leukemia (MLL) histone methyltransferase with lens epithelium‐derived growth factor (LEDGF), a chromatin‐associated protein implicated in leukemia.^(^ ^43^ ^)^ Binding of menin to MLL1 leads to upregulation of Hox gene transcription and leukemia in MLL‐rearranged acute myeloid leukemia (AML) and acute lymphocytic leukemia (ALL).^(^ ^44^ ^)^

With respect to bone, we previously reported that menin interacts with bone BMP‐2 regulated Smads, such as Smad1 and Smad5, and the osteoblast regulator, Runx2, to facilitate the commitment of mouse multipotential mesenchymal stem cells into the osteoblast lineage.^(^ ^8^ ^)^ As the committed osteoblasts continue to differentiate, menin interacts with Smad3, mediating the negative regulation of Runx2 by TGF‐β. Both BMP‐2 and TGF‐β have also been identified in human mesenchymal stem cells.^(^ ^45^ ^)^

The reduction in bone mass due to osteoclastic activity, found in the present study, is consistent with results of a recent communication examining the role of menin in bone by disrupting the *MEN1* gene at different stages of the osteoblast lineage.^(^ ^18^ ^)^ However, in that study, the mutant *Men1* created after Cre‐mediated recombination in all mouse strains only lacked exon 3 in contrast to our model, which is deficient for exons 3–8. Nonetheless, the mutant protein was not detected by Western blot. In their *Runx2‐Cre*;*Men1* ^f/f^ knockout model, the authors report unaltered osteoblast numbers or bone formation metrics, along with increased osteoclast and osteocyte densities. They present similar findings in their *Dmp1‐Cre*; *Men1* ^f/f^ osteocyte‐knockout strain as we found in our current knockout mice but could not detect differences in RANKL and OPG expression, suggesting instead that the mediator was the CLX10 chemokine. However, RANKL, which was elevated in our studies, has been established to be the major biological driver of osteoclastogenesis, and its blockade in osteoporosis, for example, is known to demonstrate potent antiresorptive action, whereas other emerging molecules may have less significant roles.^(^ ^46^ ^)^ Furthermore, denosumab, a monoclonal antibody that binds RANKL, is effective in improving BMD and lowering bone turnover in patients with primary hyperparathyroidism.^(^ ^47^ ^)^ Osteocyte RANKL is now thought to be a major driver of bone resorption,^48^, ^49^, ^50^ ^)^ and whether increased RANKL emanates from mature osteoblasts/osteocytes alone after *MEN1* reduction in early osteoblasts and/or from other osteoblastic cells remains to be determined.

Since somatic mutations of menin in other tissues have been described^(^ ^51^, ^52^ ^)^ and somatic mutations of other genes have been implicated in osteoporosis,^(53^ ^)^ it is important to know how menin deletion might affect bone turnover depending on the stage of the osteoblastic cell that is impacted. Thus, in contrast to our present results, we previously deleted menin from mature osteoblasts and osteocytes using the same floxed menin allele but using *Osteocalcin‐Cre* as the cre driver; we also overexpressed menin in the mature osteoblast.^(^ ^17^ ^)^ Osteoporotic and osteopetrotic phenotypes were observed, respectively, originating primarily from altered osteoblast activity. Osteoblast numbers and mineral apposition rate were shown to be significantly reduced in vivo in *Osteocalcin‐Cre*;*Men1*f/f mice, while primary osteoblasts showed deficient mineral apposition and alkaline phosphatase staining in vitro. Consequently, deletion of menin in more mature osteoblasts reduced osteoblasts and especially osteocytes, as well as osteoclasts, and decreased bone, although at an older age. The comparison of our present results with menin deletion in more mature osteoblasts shows that deletion of menin in progenitors has different pathogenetic consequences than deletion of menin in osteoblasts. Nevertheless, limitations of cre driver lines and developmental effects might still limit the ability to accurately ascertain *MEN1* biology in the osteoblast lineage, eg, in the current study, the molecular contribution of alterations in muscle to bone loss remain to be defined. Nonetheless, our findings using *Osteocalcin‐Cre* taken together with the present study suggest that menin has unique functions at different stages of the osteoblast lineage to regulate bone mass, acting to stimulate osteoclastogenesis and bone resorption when deleted from early osteoblast progenitors but inhibiting osteoblast function and bone formation when deleted from the mature osteoblast.

Familial hyperparathyroidism (PHPT) is a frequent manifestation of MEN1. Skeletal complications of PHPT in MEN1 appear to have an earlier onset and be more severe than in sporadic PHPT.^(^ ^54^ ^)^ In particular, it has been reported that reductions in bone mineral density in PHPT patients with MEN1 is more frequent, extensive, and progressive than in sporadic disease.^(^ ^55^ ^)^ Our findings of bone loss in heterozygous mice with menin deletion may therefore indicate that the deleterious effect on bone of the loss of menin function per se may underlie the more severe skeletal phenotype observed in patients with PHPT of MEN1. Consequently, menin may be a potential gain‐of‐function therapeutic target for the treatment of the osteoporosis observed in PHPT with MEN1, whether by genetically restoring levels of menin in bone, reversing epigenetic changes caused by loss of menin function, for example, by inhibition of histone demethylases^(^ ^56^ ^)^ or restoring downstream changes in menin signaling.^(^ ^57^ ^)^ Whether strategies aimed at enhancing menin activity in bone may also be therapeutically useful in other forms of osteoporosis remains to be determined.

## Disclosures

The authors declare that they have no conflicts of interest that could be perceived as prejudicing the impartiality of the research.

## Author Contributions

**Jad Abi‐Rafej:** Conceptualization; formal analysis; investigation; methodology; writing – original draft; writing – review and editing. **Meisam Asgari:** Conceptualization; formal analysis; investigation; methodology; writing – original draft; writing – review and editing. **Ildi Troka:** Conceptualization; formal analysis; investigation; methodology; writing – original draft; writing – review and editing. **Lucie Canaff:** Conceptualization; formal analysis; investigation; methodology; writing – review and editing. **Ahmed Moussa:** Formal analysis; investigation; writing – review and editing. **Damiano Pasini:** Conceptualization; formal analysis; project administration; resources; supervision; writing – review and editing. **David Goltzman:** Conceptualization; data curation; formal analysis; funding acquisition; project administration; resources; supervision; writing – original draft; writing – review and editing.

## Data Availability

The data that support the findings of this study are available from the corresponding author upon reasonable request.
